# Teaching Basic Surgical Skills Using a More Frugal, Near-Peer, and Environmentally Sustainable Way: Mixed Methods Study

**DOI:** 10.2196/50212

**Published:** 2023-11-15

**Authors:** Ben Smith, Christopher Paton, Prashanth Ramaraj

**Affiliations:** 1 Department of General Surgery and Urology Frimley Health Foundation Trust Frimley, Surrey United Kingdom; 2 Imperial College School of Medicine Imperial College London London United Kingdom

**Keywords:** basic surgical skills, frugal, low cost, peer-assisted learning, near-peer learning, environmentally sustainable, free, surgical education

## Abstract

**Background:**

The Royal College of Surgeons Basic Surgical Skills (BSS) course is ubiquitous among UK surgical trainees but is geographically limited and costly. The COVID-19 pandemic has reduced training quality. Surveys illustrate reduced logbook completion and increased trainee attrition. Local, peer-led teaching has been shown to be effective at increasing confidence in surgical skills in a cost-effective manner. Qualitative data on trainee well-being, recruitment, and retention are lacking.

**Objective:**

This study aims to evaluate the impact of a novel program of weekly, lunchtime BSS sessions on both quantitative and qualitative factors.

**Methods:**

A weekly, lunchtime BSS course was designed to achieve the outcomes of the Royal College of Surgeons BSS course over a 16-week period overlapping with 1 foundation doctor rotation. All health care workers at the study center were eligible to participate. The study was advertised via the weekly, trust-wide information email. Course sessions included knot tying, suturing, abscess incision and drainage, fracture fixation with application of plaster of Paris, joint aspirations and reductions, abdominal wall closure, and basic laparoscopic skills. The hospital canteen sourced unwanted pig skin from the local butcher for suturing sessions and pork belly for abscess and abdominal wall closure sessions. Out-of-date surgical equipment was used. This concurrent, nested, mixed methods study involved descriptive analysis of perceived improvement scores in each surgical skill before and after each session, over 4 iterations of the course (May 2021 to August 2022). After the sessions, students completed a voluntary web-based feedback form scoring presession and postsession confidence levels on a 5-point Likert scale. Qualitative thematic analysis of voluntary semistructured student interview transcripts was also performed to understand the impact of a free-to-attend, local, weekly, near-peer teaching course on perceived well-being, quality of training, and interest in a surgical career. Students consented to the use of feedback and interview data for this study. Ethics approval was requested but deemed not necessary by the study center’s ethics committee.

**Results:**

There were 64 responses. Confidence was significantly improved from 47% to 73% (95% CI 15%-27%; *P*<.001; *t*_13_=5.3117) across all surgical skills over 4 iterations. Among the 7 semistructured interviews, 100% (7/7) of the participants reported improved perceived well-being, value added to training, and positivity toward near-peer teaching and 71% (5/7) preferred local weekly teaching. Interest in a surgical career was unchanged.

**Conclusions:**

This course was feasible around clinical workloads, resourced locally at next to no cost, environmentally sustainable, and free to attend. The course offered junior doctors not only a weekly opportunity to learn but also to teach. Peer-led, decentralized surgical education increases confidence and has a positive effect on perceptions about well-being and training. We hope to disseminate this course, leading to reproduction in other centers, refinement, and wide implementation.

## Introduction

### Background

The Basic Surgical Skills (BSS) course is offered by all 4 UK Royal Colleges of Surgeons (RCSs). BSS is highly encouraged and is considered as compulsory at the registrar or residency stage but can be completed at the senior medical student and intern level. Surgical skill outcomes form part of both the postgraduate surgical and medical student curricula [1,2]. The achievement of these skills at UK medical schools is poor, with surgical teaching weighted toward gowning, gloving, and consultant-led theoretical teaching [3].

Although these courses are “not treated as profit making vehicles,” their high cost is underpinned by location and resource and instructor availability [4]. However, tutor qualification, regardless of previous experience, has been found to confer no difference in surgical skill performance [5]. Furthermore, 2 recent meta-analyses have demonstrated that near-peer learning (NPL) or peer-assisted learning (PAL) are very effective in teaching clinical and practical skills [6,7]. A UK surgical trainee typically spends £9105 (US $11,106.55) on courses. In the current financial climate, local courses can act as free or affordable ways to learn and consolidate skills [8].

There is evidence suggesting that local, intermittent, NPL or PAL surgical skill courses are effective, are affordable, and have high quality. A recent randomized controlled trial of 20 sessions (45 min each) led to a significant increase in surgical skill performance. This was significantly increased when taught by a near-peer tutor for knot tying, suturing, and simulated laparotomy [9]. Confidence in more advanced skills, such as arterial ligation, was demonstrated in a 4-session program using both PAL and faculty-led teaching [10]. A regional course led by senior medical students and junior doctors at a UK tertiary center also led to significant improvements in confidence across 8 skill domains [11].

Surgical education has suffered owing to the COVID-19 pandemic, with the cancellation of centralized courses, local training opportunities, and elective theater lists. The negative effect on the Annual Review of Competency Progression outcomes, training extension, and logbook completion is well documented [12]. A recent survey suggests that only 4 postgraduate surgical training posts in the United Kingdom meet the minimum quality standards [13], and the latest survey of UK surgical trainees illustrates a lack of training opportunities and the need for alternative modes of teaching [14]. Average yearly attrition from UK surgical training from 2016 to 2021 is 2.68% and has been increasing [15]. Furthermore, as affordability gains primacy as a health care metric, local, high-quality teaching represents an important surgical example of frugal innovation [16,17].

There is evidence suggesting that local PAL or NPL alternatives can be effective in teaching BSS. Although quantitative data exist, limited qualitative data exist regarding the effect of these courses on factors such as well-being, value added to training, or opinions about teaching modalities.

The objective of this study was to evaluate the impact of a novel 16-week program of weekly, lunchtime BSS sessions run at a UK district general hospital on both quantitative and qualitative factors.

### Aims

The aims of this study included the following:

1. Evaluating the effect of a novel, 10-session program of weekly BSS sessions on student confidence in each taught skill before and after the session
2. Evaluating student opinion through semistructured interview in the following domains: *opinion about the course, impact on specialty choice, value added to surgical placement, effect on well-being, opinion about PAL* and *preference of weekly versus intensive short teaching program*

## Methods

### Course Structure and Delivery

A 10-session curriculum based on the BSS course (Multimedia Appendix 1) was taught over a 16-week period, corresponding with a foundation year rotation for 4 iterations from May 2021 to August 2022. Sessions were delivered weekly for 2 hours either by a consultant, near peer, or peer tutor over lunchtime. The course structure is available in Multimedia Appendix 1.

The hospital’s canteen was contacted, and the chef kindly sourced pig skin and pork belly not suitable for sale, free of charge, as tissue models for this course. This was procured alongside the standard biweekly hospital meat delivery. A room was booked in advance at the hospital and free of charge. When unavailable, the doctors’ mess was used. Out-of-date surgical equipment was sourced from the operating theaters. Sessions on abscess drainage or cyst excision and laparoscopic skills were taught over 2 weeks.

The course was publicized to all employees of the hospital via the weekly, trust-wide emails. The decision was made to include all clinical and nonclinical staff of any grade and from any specialty, with each session being “walk-in” in style. A lead instructor was confirmed in advance from the general surgery, urology, vascular, and orthopedics junior and consultant surgeon email directory, and a lead facilitator was assigned per session from volunteer foundation doctors rotating through surgical departments.

The role of the lead instructor was to introduce the session, ensure that learning objectives were made clear and achieved, ensure that session timings were met, and assist near-peer teachers. The role of the facilitator was to ensure that session materials were obtained from the hospital canteen, a room was booked, and web-based feedback forms had been created.

Feedback was sought on a voluntary basis in a paper-free manner from all participants before and after the sessions. An example feedback form is available in Multimedia Appendix 2. Google Forms were used in conjunction with QR codes. A Google integration was used to automate the population and distribution of attendance certificates to students upon completion of the feedback form as an incentive.

### Study Design

**Figure 1:**
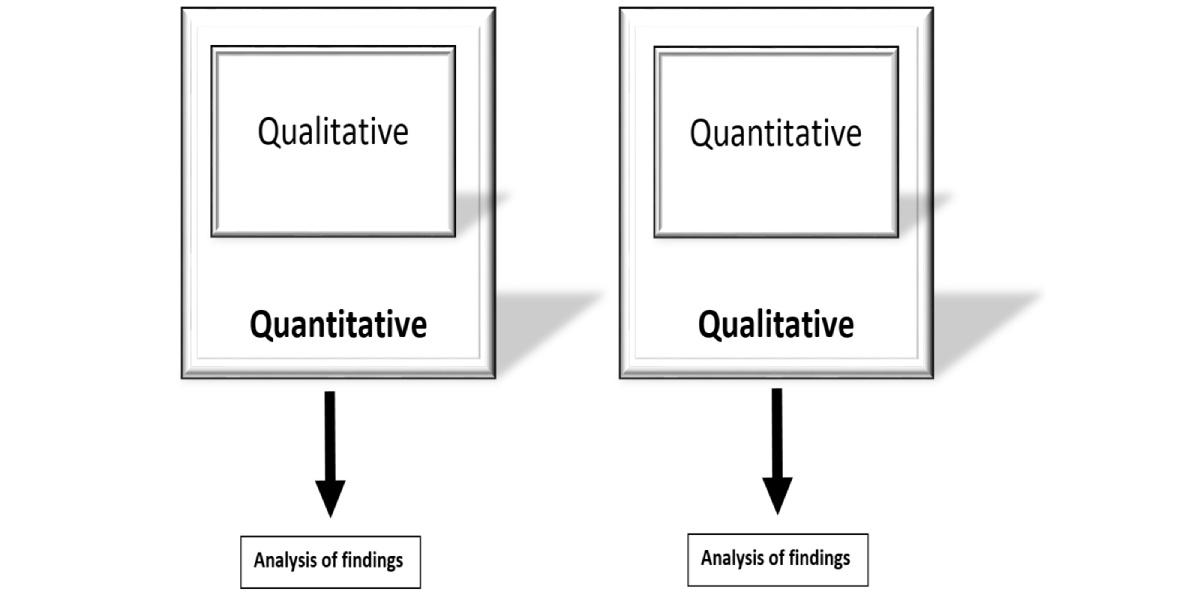
Illustration of a concurrent, nested, mixed methods design.

A mixed methods approach using a concurrent nested design as per the typology by Plano Clark and Creswell [18] and mixed methods reporting guideline recommendations was used (Figure 1) [19]. This was used to provide a broad perspective than simply using an approach and allow qualitative investigation of effects that were not previously investigated such as perceived well-being.

The purpose was to gauge the impact of the course on the workforce’s perceived confidence in surgical skills, resilience, and motivation toward a career in surgery.

### Quantitative Analysis

A Google Form (Multimedia Appendix 2) was used to collect feedback from students after each session and is outlined in the following section:

1. Grade: health care assistant, nurse, operating department practitioner, medical student, foundation year or equivalent, core trainee or equivalent, specialty trainee or equivalent, or other
2. Confidence
  Before the session: strongly disagree, disagree, neutral, agree, or strongly agree (supervised and unsupervised)
  After the session: strongly disagree, disagree, neutral, agree, or strongly agree (supervised and unsupervised)
  For those who answered either “strongly disagree” or “disagree,” there was an additional question—“How many more sessions of ‘X’ do you feel you would need to attend to become confident to perform X skill independently?”
3. Session factors
  Well structured: strongly disagree, disagree, neutral, agree, or strongly agree
  Adequate supervision: strongly disagree, disagree, neutral, agree, or strongly agree
  Materials were adequate for my learning: strongly disagree, disagree, neutral, agree, or strongly agree

Data are presented as a raw numbers and percentages. Percentages are rounded to the nearest integer.

Presession and postsession confidence analysis was conducted using the SPSS Statistics (version 28.0.1; IBM Corp) package. Percentage overall confidence was defined as the sum of the number of “confident” and “very confident” responses for “under supervision” and “without supervision” divided by the sum of the number of all responses for both “under supervision” and “without supervision.” Overall confidence across the course was calculated as the sum of overall confidence per session divided by the number of sessions. The percentage of students who stated that they were “confident” or “very confident” to perform the skill before each session was compared with the percentage of students who were “confident” or “very confident” to perform the skill after each session using the paired 1-tailed *t* test. The Shapiro-Wilk test was used to test percentage confidence for normalcy. The null hypothesis (normal distribution) was accepted for overall percentage confidence (α=.05; *P*=.24; W=0.95 where α is the probability of type 1 error), confidence to perform the skill under supervision (α=.05; *P*=.07; W=0.88), and confidence to perform the skill independently (α=.05; *P*=.98; W=0.98). The difference between the percentage of students who were “confident” or “very confident” to perform the skill before versus after each session was measured to calculate the difference across the whole course. This was performed for student confidence in performing the skills under supervision, independently, and overall. Data from laparoscopic skill sessions were not compared owing to heterogeneity in data collection among course iterations.

### Qualitative Analysis

Students were retrospectively contacted via email and asked to complete a semistructured interview covering six areas, with an open question and Likert-scale or preference-based question for each (Multimedia Appendix 3):

1. General opinion about the BSS course
2. Impact on choice of specialty and why
3. Value added to junior doctor rotation and in what way
4. Effect on well-being
5. Opinion about near-peer teaching versus faculty-led teaching
6. Preference of weekly versus consolidated program for BSS

Thematic analysis was performed by authors, BS and CP, by coding major themes and subthemes from free-text answers and quantifying the number of responses related to each theme, following guidance from literature [20]. Agreement was achieved by inclusion of themes if there was consensus between the 2 coding authors. If there was disagreement, the discrepancy was discussed among all 3 authors to achieve consensus on included themes. These data are described as raw numbers and percentages, as was the discrete data from Likert scales and preference questions.

### Ethical Considerations

The research and ethics committee of the study center was contacted to request for ethics approval. The authors were informed that ethics approval was not required as this study does not involve patient data, and all study participants provided written consent for nonidentifiable feedback data to be used for the purposes of this study. Attendance certificates were provided to students as an incentive upon the completion of the feedback form.

## Results

### Quantitative Analysis

#### Student Composition

There were 64 responses over 4 rotations, mainly from foundation doctors (n=52, 81%), followed by medical students (n=6, 9%), core surgical trainees or equivalent trust grades (n=5, 8%), and a nurse (n=1, 2%).

#### Student Confidence

Confidence results are illustrated in Table 1. A full narrative description per session is included in Multimedia Appendix 4. Confidence improved after the sessions in every surgical skill taught, for both supervised and unsupervised confidence. The largest number of responses were in the knot tying, suturing, and joint aspiration sessions. The lowest responses were in the fracture reduction, plastering, and abdominal closure sessions.

For the joint aspiration sessions, feedback was provided for individual joint (wrist and knee) in the second rotation compared with overall confidence for the session as a whole for rotations 1 and 3. There were 6 responses for confidence in wrist aspiration and 6 responses for confidence in knee aspiration, giving 12 data entries for this session. Combined with 2 responses in other rotations, in which feedback was not categorized based on joint, there were 14 total data entries.

For the laparoscopic skill session, data collection varied per rotation. Rotations 1 and 3 were measured based on skill, whereas rotation 2 was measured based on overall confidence—this is demonstrated in Table 1. The outcome for number of sessions required for confidence was omitted owing to inadequate completion.

Paired 1-tailed *t* test was used to measure the difference in confidence before and after the sessions. Across all sessions, except laparoscopic skills, which were excluded from analysis, the mean percentage of student responses in which students stated confidence to perform each skill supervised or unsupervised was 47%, whereas after the sessions, 73% of the students stated that they were confident to perform the skill supervised or unsupervised. The difference in confidence was 26% (95% CI 15%-27%; *P*<.001; *t*_13_=5.3117).

The mean percentage of students who stated that they were confident in each skill increased the most in confidence to perform the skill under supervision with 60% before the sessions and 92% after the sessions (95% CI 12%-51%; *P*=.008; *t*_13_=3.9335). The increase in confidence to perform each skill unsupervised was 20%; however, this was not statistically significant (34% before the sessions to 53% after the sessions; 95% CI 5%-44%; *P*=.11; *t*_13_=1.7435).

**Table 1:** Student confidence before and after the teaching sessions.

Sessions and categories			Response before the sessions, n (%)											Response after the sessions, n (%)								
			Strongly disagree		Disagree		Neutral		Agree		Strongly agree		Strongly disagree			Disagree		Neutral		Agree		Strongly agree
**1: Knot tying (n=10)**																						
	Supervised	0 (0)		3 (30)		1 (10)		2 (20)		4 (40)		0 (0)			0 (0)		0 (0)		5 (50)		5 (50)	
	Unsupervised	2 (20)		2 (20)		1 (10)		3 (30)		2 (20)		0 (0)			1 (10)		2 (20)		5 (50)		2 (20)	
**2: Suturing (n=15)**																						
	Supervised	0 (0)		3 (20)		2 (13)		7 (47)		3 (20)		0 (0)			0 (0)		0 (0)		6 (40)		9 (60)	
	Unsupervised	3 (20)		2 (13)		2 (13)		5 (33)		3 (20)		0 (0)			0 (0)		6 (40)		4 (27)		5 (33)	
**3: Abscess drainage and cyst excision (n=7)**																						
	Supervised	0 (0)		1 (14)		0 (0)		5 (71)		1 (14)		0 (0)			0 (0)		0 (0)		3 (43)		4 (57)	
	Unsupervised	0 (0)		2 (29)		3 (43)		1 (14)		1 (14)		0 (0)			2 (29)		2 (29)		1 (14)		2 (29)	
**4: Abdominal closure (n=5)**																						
	Supervised	1 (20)		0 (0)		2 (40)		1 (20)		1 (20)		1 (20)			1 (20)		0 (0)		0 (0)		3 (60)	
	Unsupervised	0 (0)		1 (20)		2 (40)		2 (40)		0 (0)		0 (0)			0 (0)		1 (20)		4 (80)		0 (0)	
**5: Joint aspiration (n=14)**																						
	Supervised	0 (0)		1 (7)		2 (14)		8 (57)		3 (21)		0 (0)			0 (0)		0 (0)		5 (36)		9 (64)	
	Unsupervised	1 (7)		6 (43)		5 (36)		1 (7)		1 (7)		0 (0)			1 (7)		9 (64)		3 (21)		1 (7)	
**6: Fracture reduction (n=4)**																						
	Supervised	0 (0)		1 (25)		2 (50)		0 (0)		1 (25)		0 (0)			0 (0)		0 (0)		1 (25)		3 (75)	
	Unsupervised	1 (25)		2 (50)		1 (25)		0 (0)		0 (0)		0 (0)			0 (0)		3 (75)		1 (25)		0 (0)	
**7: Plastering (n=6)**																						
	Supervised	1 (17)		1 (17)		0 (0)		2 (33)		2 (33)		1 (17)			0 (0)		0 (0)		2 (33)		3 (50)	
	Unsupervised	2 (33)		1 (17)		0 (0)		3 (50)		0 (0)		1 (17)			0 (0)		1 (17)		4 (67)		0 (0)	
**8a: Laparoscopic skills (by skill; n=3)**																						
	Graspers: move objects between graspers	0 (0)		1 (33)		0 (0)		2 (67)		0 (0)		0 (0)			0 (0)		0 (0)		2 (67)		1 (33)	
	Graspers: stack dice	0 (0)		1 (33)		0 (0)		1 (33)		1 (33)		0 (0)			0 (0)		0 (0)		2 (67)		1 (33)	
	Scissors: cut shapes	0 (0)		1 (33)		1 (33)		1 (33)		0 (0)		0 (0)			0 (0)		0 (0)		3 (100)		0 (0)	
	Reef knot	2 (67)		0 (0)		0 (0)		1 (33)		0 (0)		0 (0)			0 (0)		2 (67)		1 (33)		0 (0)	
**8b: Laparoscopic skills (procedural confidence; n=3)**																						
	Confidence in assisting laparoscopic procedures	1 (33)		0 (0)		0 (0)		2 (67)		0 (0)		0 (0)			0 (0)		1 (33)		1 (33)		1 (33)	

#### Session Factors

The results for feedback questions related to session factors are illustrated in Table 2.

Each factor demonstrated mostly positive response, with only session structure obtaining a single negative response.

**Table 2:** Participant feedback results for session factors (structure, supervision, and teaching materials).

Factor	Response, n (%)				
	Strongly disagree	Disagree	Neutral	Agree	Strongly agree
Structure: the session was well structured (n=62)	1 (2)	0 (0)	4 (6)	30 (48)	28 (45)
Supervision: I felt adequately supervised (n=62)	0 (0)	0 (0)	5 (8)	21 (34)	36 (58)
Teaching materials: the teaching materials were adequate for my learning (n=59)	0 (0)	0 (0)	4 (7)	25 (42)	30 (51)

### Qualitative Analysis

#### Semistructured Interviews

In total, 7 semistructured interviews were completed over 3 rotations. Refer to Multimedia Appendix 5 for figures outlining the full thematic analysis for each question.

#### Baseline Questions

##### Question 1: Were You Interested in Surgery as a Potential Career?

A slight majority of 57% (4/7) of attendees selected “yes,” with 43% (3/7) selecting “no” for this question.

##### Question 2: Any Thoughts on Leaving Medicine Post Qualification?

Most attendees (5/7, 71%) at baseline answered “yes.” Only 29% (2/7) had not had any thoughts about leaving medicine after qualification.

##### Question 3: What Was Your Overall Opinion of Your Surgical Rotation?

The overall opinion about surgical rotations among those surveyed was negative, with 71% (5/7) choosing “negative” and 29% (2/7) selecting “positive.”

#### Main Questions

Main questions are as follows:

- Question 1—How did you find the basic surgical skills weekly course?
- Question 2—Did attending sessions have an impact on your choice of future specialty, if so in what way?
- Question 3—Did you find the sessions added value to your surgical placement/training, if so in what way?
- Question 4—Did attending practical sessions away from the ward influence your wellbeing and if so, how?
- Question 5—What’s your opinion on peer assisted learning?
- Question 6—If you could be taught these skills during either a 16-week placement with weekly rostered sessions or in an intensive two days, which would you prefer and why?

Each question was accompanied by a Likert-scale response with the options “very negative/negative/neutral/positive/very positive” for questions 1 and 5; yes or no for question 2; “none, very little, moderately, significantly, greatly” for questions 3 and 4; and “16-weeks, neutral, 2 days” for question 6.

#### Thematic Codes and Excerpts

For question 1, a total of 5 major themes were identified: structure (1/7, 14%), usefulness (5/7, 71%), setting (2/7, 29%), availability (3/7, 43%), and content (3/7, 43%). The subthemes within “usefulness” were “practice surgical skills” (3/7, 43%) and “receiving training” (2/7, 29%). In the “availability” theme, “do not have to compete for theatre” (2/7, 29%) was most common, followed by “BSS course booked” (1/7, 14%). Regarding “content,” the main subthemes were “covers BSS” (2/7, 29%) and “felt like a trainee” (1/7, 14%). The setting theme consisted of the “outside of theatre” (1/7, 14%) subtheme, and structure was “well-structured” (1/7, 14%).

The tone of all the free-text responses was positive. Following is an example excerpt:

Good – able to learn basic skills without fighting others for theatre time.

There was only 1 free-text response to question 2, which was as follows:

More likely to choose surgery.

Overall, 3 major themes were identified for question 3: “improving surgical skills” (3/7, 43%), “personal awareness” (1/7, 14%), and “improved training” (6/7, 86%). In the “improving surgical skills” theme, 3 subthemes were identified, such as “suturing” (1/7, 14%), “confidence to go to theatre” (1/7, 14%), and “practice outside of theatre” (1/7, 14%). Regarding “personal awareness,” the specific context was “areas to improve” (1/7, 14%). In the “improved training” theme, the main subtheme was “only training received” (4/7, 57%), followed by “felt like being trained” (1/7, 14%) and “limited theatre time offered” (1/7, 14%).

Following is an excerpt:

Only departmental teaching – otherwise would be 100% service provision.

Question 4 identified 3 major themes: “setting” (5/7, 71%), “distraction” (1/7, 14%), and “sensation of learning/improving” (6/7, 86%). Regarding “setting,” the subthemes were “away from wards” (4/7, 57%) and “change on environment” (1/7, 14%). Regarding “distraction,” the context was “attention to specific skills” (1/7, 14%). Regarding “sensation of learning/improving,” the subthemes were “no other training” (4/7, 57%), “break from service provision” (1/7, 14%), and “acted as a break” (1/7, 14%).

Following is an excerpt:

Yes, by being away from the ward and paying attention to your training on specific skills you feel like you are improving and learning something.

In total, 2 major themes were identified for question 5: “enjoyable” (7/7, 100%) and “pace” (1/7, 14%). In the “enjoyable” theme, the main subtheme was “learning from others” (5/7, 71%), followed by “teaching others” (2/7, 29%). Regarding “pace,” the context was “able to go at own pace” (1/7, 14%).

Following is an excerpt:

Good to be able to learn from one another.

For question 6, coding was split among those who preferred 2 days, those who preferred 16 weeks, and those who were neutral. Among those who preferred 2 days, the main themes were “easier” (2/7, 29%) and “density of learning” (1/7, 14%). Among those who preferred 16 weeks, responses were varied: “time for improvement” (2/7, 29%), “unable to attend all” (2/7, 29%), “able to develop questions” (1/7, 14%), “selectivity” (2/7, 29%), “enjoy weekly teaching” (2/7, 29%), “consolidate skills” (1/7, 14%), and “would not attend paid course” (1/7, 14%). The placement-dependent nature of the 14% (1/7) neutral responses is outlined in the following excerpt.

An attendee highlighted how the nature of the placement could influence this preference:

Depends on the placement. If your job is going to be very theatre based then it is better to do as two days at the start. If your job is more ward based then over 16 weeks provides time to practice skills and develop better questions to ask.

Nice to be able to consolidate BSS knowledge from RCS course.

#### Analysis of the Likert-Scale Responses to Each Main Question

Attendees had a positive experience overall, with 86% (6/7) rating their experience as “very positive” and 14% (1/7) rating it as “positive.” Most attendees found that the course did not influence their choice of future specialty, with 86% (6/7) selecting “no.” Only 14% (1/7) of the participants selected “yes.” Overall, attendees found that the sessions added value, with 57% (4/7) selecting “greatly,” 29% (2/7) selecting “significantly,” and 14% (1/7) selecting “moderately.” Overall, attendees found that the sessions improved well-being, with 57% (4/7) selecting “significantly” and 43% (3/7) selecting “greatly.” Overall, attendees had a positive opinion about PAL, with 86% (6/7) rating their opinion as “very positive” and 14% (1/7) selecting “positive.” Most attendees preferred a 16-week lunchtime course, with 71% (5/7) selecting this option and 14% (1/7) selecting “neutral” and “2 days,” respectively.

## Discussion

### Principal Findings

This novel, 10-session BSS program improved confidence across all the surgical skills taught and improved student well-being through a series of structured practical sessions. The increase in student-reported overall confidence in skills taught across the course increased by 26% (95% CI 15%-27%; *P*<.001; *t*_13_=5.3117). Most preferred to be taught over a 16-week period (4/7, 57%), enjoyed peer learning or NPL (7/7, 100%), and felt that it added value to the training (6/7, 86%).

### Limitations

A limitation is that, for the joint aspiration sessions, the feedback was provided for individual joint (wrist and knee) in the second rotation compared with overall confidence for rotations 1 and 3, providing additional weighting to the second session in the results. Ideally, this would have been standardized and permitted the appreciation of joint-specific differences. Similarly, feedback for the laparoscopic skills session was incongruous owing to changes to the question posed on the feedback form between iterations. This would ideally have been standardized.

In future iterations of the course, organizers will endeavor to offer voluntary Objective Structured Assessments of Technical Skills to allow for the validation of the skills learned. The Objective Structured Assessments of Technical Skills scores could then be used to compare with those of the RCS BSS course to ensure the same standard.

In feedback-related data, there may be a degree of sampling bias as only outliers may wish to provide feedback. This was mitigated by automating the certificates of attendance once a feedback form was completed; therefore, all the students would complete the feedback forms to attain a certificate of attendance rather than just those who strongly wished to provide feedback. Self-selection bias is a feature in this cohort as only surgical workforce who were able to attend the sessions were able to provide feedback. Response bias was mitigated by anonymization of the feedback.

### Comparison With Previous Studies

A significant barrier to facilitating teaching was senior clinician availability leading to junior clinician teaching. The results demonstrate that students valued PAL and were still able to gain confidence in surgical skills, consistent with the results of studies in a medical student cohort [10]. We believe this shows that senior clinician availability should not prevent students from teaching and illustrates how peer learning or NPL can lead to better teaching outcomes, particularly for practical skills [7].

Some strengths of this course were its frugality, use of local resource, and absence of cost for the participant. Most meat products used for BSS practice were waste offcuts from hospital kitchens. In addition, we used expired sutures and scalpels from the theater department. Our feedback forms were also digitalized using QR codes, and this meant that, once generated, only minimal changes needed to be made to the forms between rotations, and no paper waste was produced. This allowed the course to be organized by junior clinicians as it required little time during the day once the automation was set up. We believe this course is a strong example of frugal, environmentally sustainable innovation within surgical education. The average cost to run each course was £5 (US $6.13) to £10 (US $12.26) for pork belly for the abdominal wall closure session. Students and educators were not required to travel across the country or further to attend a much-in-demand RCS BSS course.

The weekly nature of the course received very positive feedback, with students noting how it gave them “time for improvement” and did not penalize them if they were unable to attend all the sessions, without having to arrange study leave. Before BSS, students found the course as a useful primer, and after BSS, students found that this provided time for consolidation and an opportunity to teach colleagues.

There is good evidence to suggest that surgical skill teaching in medical school does not instill graduates with confidence in their practical skills such as suturing and knot tying [3]. This cohort has been the focus of previous studies such as that by Down et al [10], who demonstrated in a medical student cohort that BSS teaching by senior students (near peers) could improve confidence and suggested that this model could be taken forward into foundation training. When compared with our course, all 10 sessions improved student confidence in supervised procedures, consistent with previous studies evaluating the use of near-peer tutors for BSS teaching [9,10], and we have now shown how this is also applicable to a large cohort of recent graduates. In agreement with Pinter et al [9], this course also found high attendance and response rates from our knot tying and suturing sessions (10-15 students) compared with other sessions (≤7 students), which may suggest that students found the most value when practicing the frequently used BSS.

Furthermore, our use of near-peer tutors appears to initially support the work of Kim et al [5], demonstrating that the use of skill tutors can lead to similar learning outcomes compared with qualified surgeons when BSS are taught.

One of the major barriers to the RCS BSS course is the financial cost in the region of £650 (US $792.66), not inclusive of travel or accommodation costs. RCS BSS local centers price the course independently thus 1 center may charge £550 (US $670.67), whereas another may charge £850 (US $1,036.49). This novel course was free, local, and open to all clinical and nonclinical staff or students. Coupled with the increasing personal cost of surgical training, currently estimated to be >£9000 (>US $11,070) per junior doctor [8], these cost savings can make a significant difference to graduates and remove financial barriers to training.

This course will be refined and disseminated with data collected to demonstrate its effect in a large cohort. The aim is to expand this free-to-attend, low-cost, and local program to regional hospitals and collaborate nationally to try to improve the teaching of BSS. A list of recommendations for implementing and improving this course can be found at Multimedia Appendix 6.

### Conclusions

In a training landscape of expensive, infrequent courses that often require significant travel and time out of hospital, our standardized, local BSS program can provide an effective, low-cost alternative with tangible benefits regarding both training value and student well-being.

## Appendix

Multimedia Appendix 1 Structure of the course.

Multimedia Appendix 2 Example of the feedback form.

Multimedia Appendix 3 Semistructured interview.

Multimedia Appendix 4 Narrative summary of the quantitative results.

Multimedia Appendix 5 Thematic analysis of the qualitative data.

Multimedia Appendix 6 List of recommendations.

## Abbreviations

BSS: Basic Surgical Skill
NPL: near-peer learning
PAL: peer-assisted learning
RCS: Royal College of Surgeon

## Data Availability

All data generated or analyzed during this study are included in this published paper (and the multimedia appendices). The authors will endeavor to provide any further relevant data upon reasonable request via email to author BS.
