# Supplementary material for: Teaching Basic Surgical Skills Using a More Frugal, Near-Peer, and Environmentally Sustainable Way: Mixed Methods Study
Source: JMIR Perioper Med. 2023 Nov 15;6:e50212. doi: 10.2196/50212 (PMC10687689; doi:10.2196/50212)
Supplement: Multimedia Appendix 1 [file periop_v6i1e50212_app1.docx]

**Appendix 1: structure of lunchtime BSS course**

Session one: surgical knot tying

Session two: suturing

Session three: abscess drainage/cyst excision/skin lesions

Session four: abscess drainage/cyst excision/skin lesions

Session five: anterior abdominal wall closure

Session six: joint aspiration

Session seven: fractures and fracture reduction

Seven eight: plastering

Session nine: laparoscopic skills

Session ten: laparoscopic skills
