# Supplementary material for: Teaching Basic Surgical Skills Using a More Frugal, Near-Peer, and Environmentally Sustainable Way: Mixed Methods Study
Source: JMIR Perioper Med. 2023 Nov 15;6:e50212. doi: 10.2196/50212 (PMC10687689; doi:10.2196/50212)
Supplement: Multimedia Appendix 2 [file periop_v6i1e50212_app2.docx]

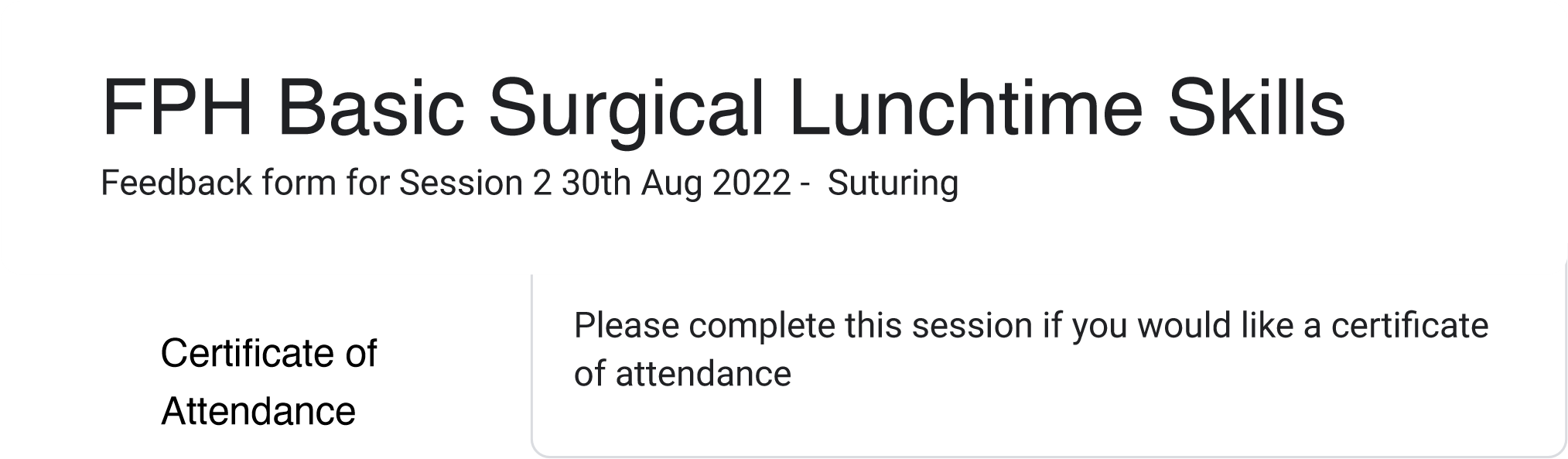


1. How would you like your name to appear on your certificate?
2. What is your grade?

*Mark only one oval.*

HCA

Nurse

ODP

Medical student

Foundation Doctor or equivalent Trust Grade

Core Training Doctor or equivalent Trust Grade (post-FY2)

Specialty Registrar or equivalent Trust Grade (ST3+)

Other:

Prior to this

session

Prior to FPH Basic Lunchtime Surgical Skills Session 2 -

Suturing.

1. I would feel confident to close skin under supervision.

*Mark only one oval.*

Strongly disagree

Disagree

Neutral

Agree

Strongly agree

1. I would feel confident to close skin independently.

*Mark only one oval.*

Strongly disagree

Disagree

Neutral

Agree

Strongly Agree

After this session

After FPH Basic Lunchtime Surgical Skills Session 2 -

Suturing.

1. I would feel confident to close skin under supervision *Mark only one oval.*

Strongly disagree

Disagree

Neutral

Agree

Strongly Agree

1. I would feel confident to close skin independently

*Mark only one oval.*

Strongly disagree

Disagree

Neutral

Agree

Strongly Agree

1. If you have answered "disagree" or "strongly disagree" to the previous question, how many more sessions of "Suturing" do you feel you would need to attend to become confident to hand tie a knot independently? (If you have answered "confident" or "very confident" to the question above, please select "0") *Mark only one oval.*

0

1

2

3

4

5

Session feedback

Please let us know about the session itself

1. The session was well structured

*Mark only one oval.*

Strongly disagree

Disagree

Neutral

Agree

Strongly agree

1. I felt adequately supervised

*Mark only one oval.*

Strongly disagree

Disagree

Neutral

Agree

Strongly Agree

1. The training materials were adequate for my learning

*Mark only one oval.*

Strongly disagree

Disagree

Neutral

Agree

Strongly Agree

1. Are there any basic surgical skills you would like to cover not yet on the list of sessions?
2. Any other feedback?

This content is neither created nor endorsed by Google.

[Forms](https://www.google.com/forms/about/?utm_source=product&utm_medium=forms_logo&utm_campaign=forms)
