# Supplementary material for: Teaching Basic Surgical Skills Using a More Frugal, Near-Peer, and Environmentally Sustainable Way: Mixed Methods Study
Source: JMIR Perioper Med. 2023 Nov 15;6:e50212. doi: 10.2196/50212 (PMC10687689; doi:10.2196/50212)
Supplement: Multimedia Appendix 3 [file periop_v6i1e50212_app3.docx]

**Appendix 3 – example of semi-structured interview form**

1. a) How did you find the basic surgical skills weekly course?

b) And if you were to rate it, how would you do so?

| Very Negative | Negative | Neutral | Positive | Very positive |
| --- | --- | --- | --- | --- |
| 1 | 2 | 3 | 4 | 5 |

1. a) Did attending the sessions have an impact on your choice of future specialty, if so in what way?

| No | Yes |
| --- | --- |
| 1 | 2 |

1. a) Did you find the sessions added value to your surgical placement/training, if so in what way?

b) How much would you say?

| None | Very little | Moderately | Significantly | Greatly |
| --- | --- | --- | --- | --- |
| 1 | 2 | 3 | 4 | 5 |

1. a) Did attending practical teaching sessions away from the ward influence your wellbeing, and if so how?

b) And if you were to rate how much?

| None | Very little | Moderately | Significantly | Greatly |
| --- | --- | --- | --- | --- |
| 1 | 2 | 3 | 4 | 5 |

1. What’s your opinion on peer assisted learning? (*People from similar social groupings, who are not professional teachers, helping each other to learn and learning themselves by teaching^1^)*

| Very Negative | Negative | Neutral | Positive | Very positive |
| --- | --- | --- | --- | --- |
| 1 | 2 | 3 | 4 | 5 |

1. If you could be taught these skills during either a 16-week placement with weekly rostered sessions or an intensive two days, which would you prefer and why?

| 16-weeks | Neutral | 2 days |
| --- | --- | --- |
| 1 | 2 | 3 |

Baseline before attending the basic surgical skills course:

1. Were you interested in surgery as a potential career? Y/N
2. Any thoughts on leaving medicine post qualification? Y/N
3. What was your overall opinion of your surgical rotation? Pos/Neut/Neg

I consent to my answers being recorded and sent digitally, with the data erased once post-hoc transcription is complete in line with GDPR. In addition, I consent to my responses being anonymised and published.

Signed…………………………………. Date………………….

Name…………………….... Grade………... Date……………
