# Supplementary material for: Teaching Basic Surgical Skills Using a More Frugal, Near-Peer, and Environmentally Sustainable Way: Mixed Methods Study
Source: JMIR Perioper Med. 2023 Nov 15;6:e50212. doi: 10.2196/50212 (PMC10687689; doi:10.2196/50212)
Supplement: Multimedia Appendix 4 [file periop_v6i1e50212_app4.docx]

**Appendix 4: full narrative summary of confidence results**

**Session 1: knot tying**

There were ten responses over the three rotations.

*Prior*

Prior confidence under supervision was rated primarily as ‘*Strongly agree’* (n=4, 40%) followed by *‘Disagree’* (n=3, 30%) then *‘Agree’* (n=2, 20%) and *‘Neutral’* (n=1, 10%). The majority (n=6, 60%) were in a positive category of either *‘Agree’* or *‘Strongly agree’.*

Independent confidence was 50% positive with 30% selecting *‘Agree’* (n=3) and 20% selecting *‘Strongly agree’* (n=2). Like supervised confidence, one participant selected *‘Neutral’.* There were 40% negative ratings, split between *‘Strongly disagree’* (n=2, 20%) and *‘Disagree’* (n=2, 20%).

*After*

Post session confidence under supervision was 100% positive with 50% selecting *‘Agree’* and 50% selecting *‘Strongly agree’ (*n=5 respectively). Unsupervised confidence also improved with 70% positive, 50% selecting *‘Agree’* (n=5) and 20% ‘*Strongly agree’* (n=2). There was 20% (n=2) ‘*Neutral’* confidence and 10% selected *‘Disagree’* (n=1).

**Session 2: suturing**

There were fifteen responses over three rotations.

*Prior*

The majority (n=10, 67%) selected a positive rating of confidence under supervision; mainly *‘Agree’* with 47% (n=7) and 20% selecting *‘Strongly agree’* (n=3). There was 20% who selected *‘Disagree’* (n=3) and 13% selected *‘Neutral’* (n=2). Independent confidence had a slightly reduced majority of positive ratings; 33% (n=5) in *‘Agree’* and 13% (n=2) in *‘Strongly agree’.* There were 5 total negative confidence ratings split between 20% in *‘Strongly disagree’* (n=3) and 13% in *‘Disagree’* (n=2) with 13% also selecting *‘Neutral’* (n=2).

*Post*

Under supervision 100% of attendees selected a positive confidence rating with 60% (n=9) selecting *‘Strongly agree’* and 40% (n=6) selecting *‘Agree’.* Independent confidence also improved with 60% positive ratings (n=9) split between 33% selecting *‘Strongly agree’* (n=5) and 27% (n=4) selecting *‘Agree’*. There were no negative confidence ratings and 40% (n=6) selected *‘Neutral’.*

**Session 3: abscess drainage/cyst removal**

There were seven responses across three rotations.

*Prior*

Confidence under supervision was 86% positive (n=6), 71% being ‘agree’ (n=5) and 14% ‘strongly agree’ (n=1). There was a 14% ‘disagree’ response (n=1). Independent confidence was more dispersed; a majority of 43% (n=3) stated ‘neutral’, then 29% ‘disagree’ (n=2) and 14% (n=1) for each of ‘disagree’ and ‘agree’.

*After*

Confidence under supervision after teaching was 100% positive, split between 57% ‘strongly agree) (n=4) and 43% ‘agree’ (n=3). Independent supervision showed a slight improvement with the majority of responses being positive, 14% ‘agree’ (n=1) and 29% ‘strongly agree’ (n=2) with 29% selecting ‘neutral’ and ‘disagree’ respectively (n=2).

**Session 4: abdominal wall closure**

There were five responses across three rotations.

*Prior*

Prior confidence under supervision was 20% ‘strongly disagree’ (n=1), 40% ‘neutral’ (n=2), and 20% (n=1) respectively for ‘agree’ and ‘strongly agree’. Independent confidence was 40% ‘agree’ (n=2), 40% ‘neutral’ (n=2) and 20% ‘disagree’ (n=1).

*After*

Following teaching 60% were ‘strongly agree’ (n=3) with 20% (n=1) respectively choosing ‘disagree’ and ‘strongly disagree’. Independent confidence was rated at 20% ‘neutral’ (n=1) and 80% ‘agree’ (n=4).

**Session 5: joint aspiration**

There were nine respondents across three rotations. In the second rotation, feedback was by individual joint (wrist and knee). There were six responses so there are twelve data entries for this session. Combined with two responses in other rotations, in which feedback was not granulated by joint, there are fourteen total data entries.

*Prior*

Confidence under supervision prior to teaching was positive with 57% (n=8) answering ‘agree’ and 21% (n=3) answering ‘strongly agree’. Neutral responses were smaller with 14% (n=2) and 7% answered ‘disagree’ (n=1). Independent confidence was primarily negative with 43% (n=6) answering ‘disagree’ and 7% (n=1) answering ‘strongly disagree’. The next largest group was neutral with 36% (n=5) and 7% (n=1) selecting ‘agree’ and ‘strongly agree’.

*After*

Confidence under supervision improved with 36% (n=5) selecting ‘agree’ and 64% (n=9) selecting ‘strongly agree’. Independent confidence also improved with most attendees answering ‘neutral’ with 64% (n=9), 21% selecting ‘agree’ (n=3) and 7% selecting ‘strongly agree’ (n=1). One selected ‘disagree’ (7%, n=1).

**Session 6: fracture/joint reduction**

There were 4 total attendees over three rotations.

*Prior*

Confidence under supervision was rated as 25% ‘disagree’ (n=1), 50% ‘agree’ (n=2) and 25% ‘strongly agree’ (n=1). Independent was primarily negative with 25% ‘strongly disagree’ (n=1), 50% ‘disagree’ (n=2) and 25% ‘neutral’ (n=1).

*After*

Confidence under supervision improved following teaching with 25% choosing ‘agree’ (n=1) and 75% selecting ‘strongly agree’ (n=3). Independent confidence improved but was primarily ‘neutral’ with 75% selecting this option (n=3) and 25% selecting ‘agree’ (n=1).

**Session 7: plastering**

There were 6 total attendees over three rotations.

*Prior*

Prior confidence under supervision was primarily positive (67%), split equally with 33% (n=2) selecting ‘agree’ and ‘strongly agree’. For ‘disagree’ and ‘strongly disagree’ this was selected by 17% (n=1) respectively. Independent confidence was scored as 33% ‘strongly disagree’ (n=2), 17% ‘disagree’ (n=1) and 50% ‘agree’ (n=3).

*After*

After teaching confidence under supervision improved with 50% selecting ‘strongly agree’ (n=3_ and 33% selecting ‘agree’ (n=2). Only one attendee rated their confidence in this area as ‘strongly disagree’) (17%, n=1). Independent confidence also improved with 67% (n=4) selecting ‘agree’ and 17% (n=1) respectively selecting ‘neutral’ or ‘strongly disagree’.

**Session 8a: laparoscopic skills – analysed by skill *(see confidence table)***

There were three attendees over three rotations.

***Prior***

The majority felt mixed positive and negative confidence in the four skills taught in this session (see Table 1). Stacking dice with a grasper has the highest confidence rating with 33% (n=1) selecting ‘disagree’, 33% (n=1) selecting ‘agree’ and 33% (n=1) selecting ‘strongly agree’. Moving objects between graspers was the second most highly rated skill for prior confidence with 33% (n=1) selecting ‘disagree’, and 67% (n=1) selecting ‘agree’. Cutting shapes with scissors was the third with 33% (n=1) selecting *‘*disagree’, ‘neutral’ and ‘agree’ respectively. The lowest rated skill was tying an intracorporeal reef knot with 67% (n=2) selecting ‘strongly disagree’ and 33% (n=1) selecting ‘agree’.

***After***

Confidence improved in all four skills. Moving objects between graspers and stacking dice with graspers were both rated as 67% (n=1) ‘agree’ and 33% (n=1) ‘strongly agree’. Cutting shapes with scissors was rated as 100% (n=3) ‘agree’. Tying an intracorporeal reef knot was rated as 67% (n=1) ‘neutral’ and 33% (n=1) ‘agree’.

*Continued on next page*

**Session 8b: laparoscopic skills – overall procedural confidence**

There were three attendees over three rotations.

***Prior***

Prior confidence was mixed with 33% (n=1) selecting ‘strongly disagree’ and 67% (n=2) selecting ‘agree’.

***Post***

Confidence improved with 33% (n=1) selecting ‘neutral’, ‘agree’ and ‘strongly agree’ respectively.
