# Supplementary material for: Teaching Basic Surgical Skills Using a More Frugal, Near-Peer, and Environmentally Sustainable Way: Mixed Methods Study
Source: JMIR Perioper Med. 2023 Nov 15;6:e50212. doi: 10.2196/50212 (PMC10687689; doi:10.2196/50212)
Supplement: Multimedia Appendix 5 [file periop_v6i1e50212_app5.docx]

**Appendix 5 – semi-structured interview thematic analysis**

**Question 1 – how did you find the basic surgical skills weekly course?**

| **Q1** | |
| --- | --- |
| **Structure** | 1 |
| well-structured | 1 |
| **Usefulness** | 5 |
| practice surgical skills | 3 |
| receiving training | 2 |
| **Setting** | 1 |
| outside of theatre | 1 |
| **Availability** | 3 |
| do not have to compete for theatre | 2 |
| BSS courses booked | 1 |
| **Content** | 3 |
| covers BSS | 2 |
| felt like trainee | 1 |

**Figure 1: Thematic coding for Q1**

The major themes are highlighted in bold with sub-themes in italic below. Usefulness was the most commonly mentioned major theme followed by content and availability. The most common minor theme was ‘practice surgical skills’ relating to usefulness.

**Question 2 – no table (only one free text response)**

**Question 3 – did you find the sessions added value to your surgical placement/training, if so in what way?**

**Figure 2: Thematic coding for Q3**

The major themes are highlighted in bold with sub-themes in italic below. Improved training was the most commonly mentioned major theme, with ‘only training received’ the most common sub-theme within this. Improved surgical skills was the second most common major theme for this question, spread equally between the three sub-themes for this question.

| **Q3** | |
| --- | --- |
| **Improved surgical skills** | **3** |
| suturing | 1 |
| confidence to go to theatre | 1 |
| practice outside of theatre | 1 |
| **Personal awareness** | **1** |
| areas to improve | 1 |
| **Improved training** | **6** |
| only training received | 4 |
| felt like being trained | 1 |
| limited theatre time offered | 1 |

Continued on the next page

| **Q4** | |
| --- | --- |
| **Setting** | **5** |
| Change of environment | 1 |
| Away from wards | 4 |
| **Distraction** | **1** |
| attention to specific skills | 1 |
| **Sensation of learning/improving** | **6** |
| break from service provision | 1 |
| Acted as a break | 1 |
| No other training | 4 |

**Question 4 – did attending practical sessions away from the ward influence your well-being and If so, how?**

**Figure 3: Thematic coding for Q4**

The major themes are highlighted in bold with sub-themes in italic below. Sensation of learning/improving was the most common major theme, with ‘no other training’ the most common sub-theme within this. Setting was the next most common major theme and being away from the wards was the most common sub-theme within this.

**Question 5 – what’s your opinion on peer assisted learning?**

| **Q5** | |
| --- | --- |
| **Enjoyable** | 7 |
| learning from others | 5 |
| teaching others | 2 |
| **Pace** | **1** |
| able to go at own pace | 1 |

**Figure 4: Thematic coding for Q5**

The major themes are highlighted in bold with sub-themes in italic below. The most common major theme in this category was the concept of the sessions being enjoyable most mentioned and the most common sub-theme within this was learning from others.

continued on next page

**Question 6 – if you could be taught these skills during either a 16-week placement with weekly rostered sessions or in an intensive two days, which would you prefer and why?**

**Figure 5: Thematic coding for Q6**

Responses are divided between free-text responses based on the choice made by the participant. For the preference of a 2-day course, the main reason was this was easier. The majority preferred the 16-week model with a wide range of reasons. There was only once response for neutral and this was based on the nature of the placement a participant was on at the time.

| **Q6** | |
| --- | --- |
| 2-day | |
| easier | 2 |
| density of learning | 1 |
| 16-week | |
| time for improvement | 2 |
| unable to attend all sessions | 2 |
| able to develop questions | 1 |
| selectivity | 2 |
| enjoy weekly teaching | 2 |
| consolidate skills | 1 |
| would not attend paid course | 1 |
| Neutral | |
| Depends on placement | 1 |
