# Supplementary material for: Teaching Basic Surgical Skills Using a More Frugal, Near-Peer, and Environmentally Sustainable Way: Mixed Methods Study
Source: JMIR Perioper Med. 2023 Nov 15;6:e50212. doi: 10.2196/50212 (PMC10687689; doi:10.2196/50212)
Supplement: Multimedia Appendix 6 [file periop_v6i1e50212_app6.docx]

**Appendix 6 – recommendations to standardise and disseminate**

Establish these **before**

1. **Syllabus:** *what sessions will you run? In what order? –* we advise following the structure outlined in Appendix 1 but this can be modified.
2. **Room:** *what rooms will you use? Can you block book?* – we advise finding a way to block book a room on a rolling basis if possible for continuity.
3. **Staff:** *who will set up/pack away? Who will teach?* – we advise creating an instructor/facilitator availability group and a **core team** to help run the course.
4. **Digital infrastructure:** *where will you store information for the course?* – we advise creating a easily repeatable file infrastructure *(e.g on Google Drive)* to easily administrate the course.
5. **Feedback and certification:** *how will you collect feedback? How can you make certification easy? –* we advise creating standard templates for which you can change the date on and use the *‘CertifyMe’* software to automate this process.
6. **Validation:** *how will you validate the course?* – we advise implementing voluntary Objective Structured Assessment of Technical Skills for each session so that students can receive scores from which they can measure improvement, and for a comparator for this course with other courses.
7. **Publicity:** *how will you spread the message? Which groups are you trying to reach? –* try and get in touch with the admin team at your hospital to circulate advertisements to the wider MDT.
8. **Resources:** *where will you get animal models from? Do you have simulators? –* we recommend contacting the hospital kitchen to source waste meat, and the operating theatres for out of date equipment.

Establish these **during**

1. **Redundancy in the system:** be dynamic and flexible, try to recruit additional members of team so in the worst case someone sets up, someone facilitates and someone tidies up!
2. **Links with surgical team:** as awareness of the course grows, try to deepen links with the various surgical firms, theatre staff, etc., to improve relations and running of the course.

Establish this to **analyse**

1. **Endgame:** *what sort of research are you trying to produce?* – before you create your feedback forms, think through what data you want to analyse and standardise this to prevent incongruent results later on. We suggest following our datapoints for standardisation and data collection on a wider scale.
